# Establishment of xenografts of urological cancers on chicken chorioallantoic membrane (CAM) to study metastasis

**DOI:** 10.1093/pcmedi/pbz018

**Published:** 2019-10-01

**Authors:** Junhui Hu, Moe Ishihara, Arnold I Chin, Lily Wu

**Affiliations:** 1 Department of Molecular and Medical Pharmacology; 2 Department of Urology; 3 Department of Pediatrics; 4 Jonsson Comprehensive Cancer Center, David Geffen School of Medicine, University of California at Los Angeles, CA 90095, USA

**Keywords:** chorioallantoic membrane, prostate cancer, bladder cancer, kidney cancer, animal mode

## Abstract

Cancer of the urological system commonly occurs in the kidney, bladder, and prostate gland. The clear cell subtype of renal cell carcinoma (ccRCC) constitutes the great majority of kidney cancer. Metastatic ccRCC portends a very poor outcome with no effective treatment available. Prostate cancer is the most common cancer in males in the US. Despite recent advances in selective kinase inhibitors and immunotherapies, the rate of developing new treatment from bench to bedside is slow. A time-consuming step is at the animal drug testing stage, in which the mouse model is the gold standard. In the pursuit to streamline the *in vivo* cancer biology research and drug development, we explored the feasibility of the chicken chorioallantoic membrane (CAM) model to establish xenografts. The CAM model greatly shortens the time of tumor growth and lowers the cost comparing to immunocompromised mice. We generated CAM xenografts from ccRCC, bladder and prostate cancer, with established cancer cell lines and freshly isolated patient-derived tissues, either as primary tumor cells or small pieces of tumors. The successful CAM engraftment rate from the different tumor sources is 70% or above. Using our previously established metastatic ccRCC mouse model, we showed that the CAM xenograft maintains the same tumor growth pattern and metastatic behavior as observed in mice. Taken together, CAM can serve as a valuable platform to establish new patient-derived xenografts (PDXs) to study tumor biology, thus accelerating the development of individualized treatment to halt the deadly metastatic stage of cancer.

## Introduction

Urological malignancies frequently arise from the epithelial cells of the major organs, including the kidney, ureter, bladder, urethra, prostate, and testes^1^. Prostate cancer is the most prevalent urological cancer in males in the US, estimated to have 174 650 newly diagnosed cases and 31 620 deaths in 2019^2^. Bladder cancer is the sixth most common cancer in the US, as estimated 80 470 and 17 670, new cases and deaths, respectively in 2019^2^. As major organs involved in excretory function, cancers of the bladder and kidney are heavily influenced by environmental and carcinogen exposures, such as tobacco smoking. Interestingly, both of these malignancies are 2 to 3 times more prevalent in men than women. Despite advances in surgical technology and drug development, the survival rate of bladder cancer remained unchanged from 2009 to 2015^2^. The incidence of kidney cancer is slightly lower than bladder cancer in the US, with new cases and deaths in 2019 estimated to be 74 000 and 15 000, respectively. However, the incidence of kidney cancer is increasing in the last 20 years, from about 10 to 16.1 per 100 000 persons^2^. The clear cell histological subtype of renal cell carcinoma (ccRCC) is the most common type of kidney cancer. Although organ-confined ccRCC has a favorable 5-year survival of 74.8%, approximately 30% of patients will develop a metastatic disease with a very poor 5-year survival of only 10%. Disseminated metastatic disease is the lethal stage for all solid tumors, including all these three urological cancers. Unfortunately, no effective anti-metastasis treatment is available at this time. Further investigation of the cancer biology and testing of new therapeutics in new patient-derived tumor models is sorely needed to propel the next wave of advancement for these urological cancers^3–5^.

The mouse model has been the gold standard for studying human diseases for several decades. The reasons for the popularity of mice include their small size, ease of colony expansion, their mammalian physiology, and most importantly, the advent of transgenic engineering technology to mimic human diseases^6^. However, mouse experimentations have several limitations. First, genetically modified immunodeficient mouse strains needed for establishing patient-derived xenografts (PDXs) are very costly; many of them cost over $100 per animal. Subcutaneous heterotopic implantation is the preferred site of initial attempts of PDXs engraftment in mice, mainly due to its superficial location as the deeper location of urological organs is difficult to assess engraftment. However, the relatively poor blood supply in subcutaneous tissue can slow or prevent the engraftment process. Generally, the engraftment of new PDXs of urological cancer in mice will require at least 2 months. Tumor models established on the chorioallantoic membrane (CAM) of chicken embryo offers several advantageous over the mouse model. In general, each fertilized egg costs less than US $2, which is 1%-2% the cost of each immunocompromised mouse. The maximal time of tumor growth on CAM is 2 weeks. Moreover, the open window on the eggshell created to drop the CAM for tumor implantation also allows for direct visualization of tumor growth.

CAM is a transparent membrane that serves as the lining of allantois^7^ and extends from the ventral wall of the endodermal hind-gut of the chicken embryo^8^. The growth of this membrane starts from embryonic development day 3 in chickens^8^. It has known to provide rich vasculature and a rapidly expanding area. The use of chicken as an experimental model for cancer research initiated the era of molecular oncology. More than a century ago in 1911, Dr. Peyton Rous discovered Rous sarcoma virus (RSV) as the causative agent of chicken sarcoma^9^, and in the following year, Dr. James Murphy further demonstrated that rat sarcoma could be transplanted into the chick embryo ^10^. Later in the 1930s, CAM was frequently used to cultivate vaccines, viruses, and bacteria^11^^,^^12^. In the last few decades, CAM utilization correlated closely with the growth of angiogenesis research as CAM was shown to be a good substitute for more expensive and laborious angiogenesis assay in mammalian animals, such as the corneal pocket assay^13–15^. The application of CAM in cancer research gained traction in early 2000, coincided with the advancement in 3D and live cell and tissue imaging that can be directly applied to CAM tumor models. The use of CAM has continued to increase in the last decade as it has been shown to be a good growth platform for a wide range of cancer cell lines, such as ovarian cancer^16^, colon cancer^17^, sarcoma^18^, kidney cancer^19^, melanoma^20^, multiple myeloma^21^ and cancer tissues from hepatocellular carcinoma^22^, sarcoma^23^, melanoma^24^, and ovarian adenocarcinoma^25^.

Despite the prolific use of CAM in cancer research in recent years, few studies have assessed the ability to establish new PDXs of urological cancers from different sources of patient-derived cancer cells and tissues. Here we demonstrated that CAM PDXs can be established efficiently from pre-existing human cancer cell lines, and primary tumor cells and small tumor pieces freshly isolated from surgical samples of urological cancers. Metastasis is a frequent and deadly manifestation of ccRCC in the clinic. Here, we demonstrated that the growth and metastatic behavior of a murine ccRCC model we recently developed^26^^,^^27^ could be fully reproduced in the CAM model. Our results support that the CAM model could be a valuable alternative *in vivo* model to establish new PDXs and study the biology of urological cancers.

## Methods and Materials

### Antibodies, primers, cell lines, and reagents

Anti-FLAG antibody was purchased from eBioscience (Cat#14-6681-82), anti-panCK antibody from Biogenex (Cat#AM273-5 M), anti-VHL antibody from Abcam (Cat#ab140989), and anti-CK8/18 from Novus (Cat#NBP2-44929). Murine ccRCC cell line RENCA and human ccRCC cell line ACHN, prostate cancer cell lines CWR22v1 and C4-2 were purchased from the American Type Culture Collection (ATCC) and maintained in RPMI-1640, supplemented with 10% fetal bovine serum and penicillin/Streptomycin at a working concentration of 100 U/mL. Murine prostate cancer cells Myc-CaP were purchased from ATCC and maintained in DMEM, supplemented with 10% fetal bovine serum and penicillin/Streptomycin at a working concentration of 100 U/mL. Human bladder cancer cell lines T24 and HT-1376 were kind gifts from Dr. Arnold I. Chin and Dr. Hanwei Zhang at UCLA and maintained in the same condition as RENCA cells.

Lentiviral plasmid encoding mStrawberry and EGFP, together with flag tag or HA, and plasmid encoding firefly luciferase were constructed based on pSicoR (Addgene, #11579), and lentivirus was packaged as mentioned previously in the report^26^.

### Human ccRCC and bladder cancer patient specimen

The collection of patient ccRCC and bladder cancer tissues was undertaken according to the protocol approved by the UCLA Institutional Review Board. Clinical data, such as age, gender, and Eastern Cooperative Oncology Group performance status (ECOG PS), and pathological data, such as tumor-node-metastasis stage, histologic subtype, and Fuhrman grade, were collected from these cases. All involved patients consented to participate in the study before surgery. All experiments were performed according to the approved guidelines, complying with the principles for the use of human tissues, as stated in the Declaration of Helsinki. This study was approved by the Institutional Review Board of UCLA, under protocol # IRB 11-001363.

### Establishment of a primary cancer cell line from patient ccRCC tissues

Patient’s ccRCC samples were mechanically digested by mincing and chopping, followed by chemical digestion with Liberase (Cat#5401119001, Sigma Aldrich) at a working concentration of 0.5 u/mL in RPMI-1640. The samples were incubated in Liberase for 1 hour at 37 °C in a rotary mixer. The digestion was halted by the addition of RPMI-1640 supplemented with 10% FBS, and cells were centrifuged at 300 × *g* for 5 min to pellet. Red blood cell lysis was performed when necessary (Cat#555899, BD). Then the cells were cultured in a 15-cm dish with 20 mL of RPMI-1640 supplemented with 10% FBS and 100 u/mL Penicillin/Streptomycin.

### CAM xenograft model from cells and tissues

All experiments performed in fertilized eggs and embryo before hatching do not require IACUC approval. The CAM xenograft model was established and studied according to the previously published protocols^28^^,^^29^. Briefly, freshly laid fertilized eggs were purchased (Rhode Island Red Rooster, AA Lab Eggs). After 7 days of pre-incubation at 37-38 °C and 55%-65% humidity, the CAM beneath the lateral side of the egg shell was separated and retracted from the shell and then the overlying shell was removed to form a window for tumor implantation^28^^,^^29^. On embryonic developmental day 10, the pre-existing cancer cell lines and patient-tissue-derived primary cancer cell lines were implanted on the CAM at the concentration of 2 × 10^6^ cells/egg suspended in diluted Matrigel (Cat# 356234, Corning, USA; 1:2 diluted in pre-cooled RPMI-1640).

Tumor growth was recorded every other day, starting on tumor day 3 (or developmental day 13). For CWR22Rv1 tumors, BLI was also performed to record tumor (developmental day 19). The procedures were described in our previous reports^26^^,^^27^, except that 100 μL luciferin reconstituted to 30 mg/ml in saline and applied directly over the tumor, and 10 μL isoflurane were directly injected into the allantois with an insulin syringe. At the endpoint (developmental day 20), the embryos were euthanized by being placed on ice for 20 min. The CAM tumors were harvested for gross picture and histological analyses. Chicken blood and organs were also collected to detect metastasis.

For patient samples, tumor tissue was chopped into small chunks around 2-3 mm in diameter and put on the CAM. 200 μL diluted Matrigel was added to cover the samples for short-term nourishment. Other steps were performed as mentioned above.

For the experiment using CAM model to assess RENCA tumor growth and metastasis, 21 fertilized eggs were randomly divided into 3 groups and were implanted with VHL-WT, VHL-KO or a 1:1 mixture of both cells at 2 × 10^6^ cells/egg (*n* = 7 per group). The tumors and embryo blood were harvested at developmental day 20 and assessed by tumor weight and circulating tumor cells by flow cytometry or RT-PCR to detect mStrawberry+ or EGFP+ cells, respectively. Both VHL-WT and VHL-KO cells were also tagged by FLAG epitope to allow histological detection.

### Analysis of distant metastasis of ccRCC in hatched chicken and mouse

All animal studies described here have been approved by IACUC, designated as UCLA Chancellor’s Animal Research Committee (ARC). The chicken embryos bearing CAM tumors were allowed to hatch and grow for 2 weeks. The VHL-WT cells were tagged with HA epitope and the VHL-KO cells were tagged with FLAG epitope to facilitate histological detection. After euthanasia with isoflurane inhalation followed by cervical dislocation, the lungs were extracted and fixed in 4% paraformaldehyde for paraffin-wax embedding. The ARC 2017-102-01A protocol covered these chicken experiments. The methods of establishing orthotopic renal tumors have been described in previous studies^26^^,^^27^^,^^30^ and approved in the ARC 2002-049-53 protocol.

### Flow cytometry and immunofluorescence staining

Flow cytometry was performed on chicken blood to detect circulating tumor cells, as described previously^26^^,^^27^. Immunofluorescence staining was performed in the same way as immunohistochemical staining previously reported in our study^26^, except for TSA staining. TSA kit (Cat# NEL756001KT, PerkinElmer) was used at a 1:200 dilution ratio for tertiary signal amplification of FLAG.

### Statistics

Each experiment was performed at least in triplicates unless otherwise stated. Data are presented as mean ± standard deviation (SD). Significance was determined by a paired Student’s T-test when there were two groups or by a one-way ANOVA when there were three or more groups (GraphPad Prism ver6.0). A *p*-value cutoff of 0.05 was used for significance.

## Results and Discussion

### High-efficiency CAM engraftment with established renal, bladder and prostate cancer cell lines

**Figure 1:**
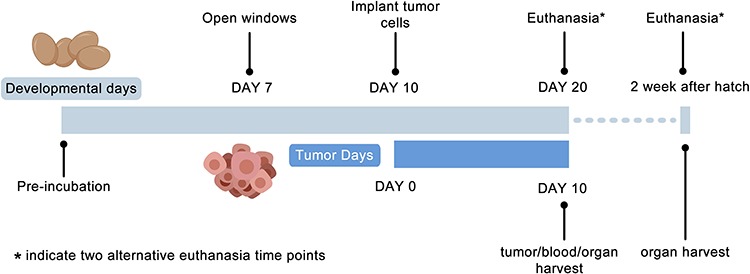
Schematic diagram of CAM xenograft implantation strategy.

**Figure 2:**
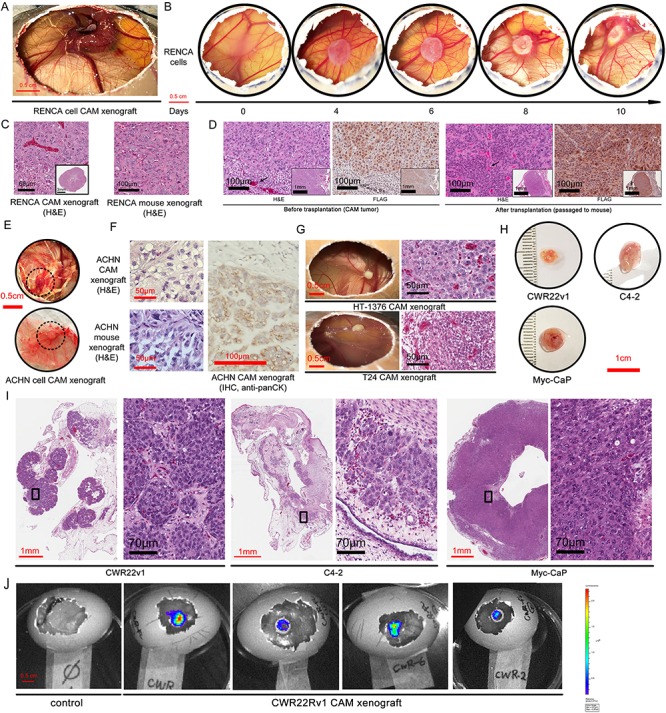
Establishment of CAM xenograft from pre-existing urological cancer cell lines. CAM tumor developed by implantation of 2 × 10^6^ murine ccRCC RENCA cells. A. A gross view of CAM tumor on embryonic day 21. B. The development of the RENCA CAM tumor over the 10 days period after the implantation of the matrigel cell suspension. C. H&E stain of the RENCA CAM xenograft in parallel with the RENCA tumor established in the mouse kidney. D. H&E and FLAG IHC staining in both the RENCA CAM tumors and the CAM tumors re-transplanted subcutaneously in the nude mice. E. Gross view and F. H&E and anti-panCK IHC staining of CAM xenograft from human ccRCC cell line ACHN. Dash circled areas are tumors. G. Gross view of CAM xenograft and H&E stained tumor section from human bladder cancer cell line HT-1376 and T24. H. Gross view and I. H&E staining of CAM xenograft from human prostate cancer cell line CWR22Rv1 and C4-2, and murine prostate cancer cell line Myc-CaP. J. With lentiviral mediated transduction of firefly luciferase gene into CWR22Rv1 cells, their CAM xenograft can be visualized by bioluminescence imaging (BLI).

The approach we have taken to establish CAM xenografts is illustrated in Fig. 1. In 2013, Fergelot *et al.* introduced human ccRCC cell lines RCC4, Caki-2 and 786-O into CAM and found that Caki-2 and 786-O formed tumors^28^. Given the short 10 days of growth in CAM, we surmise that CAM could be more favorable to support the engraftment of the faster proliferative cell lines such as the murine RENCA ccRCC cell line, which has not been assessed in previous CAM studies. As shown in Fig. 2A and 2B, RENCA cells induced angiogenesis and grew to about 1 cm in diameter within 10 days. The cellular morphology of the CAM RENCA tumor was similar to mice tumor as assessed by H&E stain (Fig. 2C). The serial passage of CAM tumors had been reported previously^24^. Here, we assessed whether CAM tumors could be passaged back in mice as a means to extend the tumor growth period. CAM RENCA tumor was re-transplanted into the subcutaneous tissue of nude mice and grew to a 1 cm diameter tumor in 3 weeks (Fig. 2D). The RENCA tumor cells were marked by the FLAG epitope. The RENCA CAM tumor before and after passaged back in mice showed a high degree of resemblance in cell morphology and FLAG expression as assessed by H&E and IHC stain, except that the chicken stroma and blood components (nucleated RBC) were replaced by the mouse counterparts (Fig. 2D, arrows). Next, we implanted the VHL-expressing human ccRCC cell line ACHN on CAM. ACHN cells established CAM xenografts consistently, but in smaller size than RENCA CAM tumors (Fig. 2E). The cell morphology of ACHN tumor engrafted in CAM and mouse was also similar (Fig. 2F). The tumor cells in the ACHN CAM tumor were further confirmed to be of human origin by a human pan-cytokeratin stain (Fig. 2F, right panel).

In comparison to human kidney and prostate cancer, bladder cancer appears to be amenable to *in vitro* cultivation. This point is reflected by a high number of distinct bladder cancer cell lines reported in publications and the availability of 10 verified bladder cancer lines in reputable repositories. In contrast, there are only 5 to 6 established human prostate or kidney cancer cell lines that are widely used in research. The experience in CAM also supports the relative ease to establish CAM xenografts from a wide range of established bladder cancer cell lines. In 2007, Chin *et al.* established the CAM xenograft with the MGH line as an *in vivo* model for fluorescence diagnosis^31^. In the last 10 years, numerous studies have employed CAM xenograft from bladder cancer cell lines such as HT1197, 639 V, RT112, KU7, UMUC2, VM-CUB1, 5647, RT112 and T24 to investigate the involvement of different pathways such as CDK4/6, PI3K, AKT and *de novo* purine metabolism in bladder cancer progression^5^^,^^32^^,^^33^. In our pilot studies, we found that both T24 and HT-1376 cells could establish xenografts consistently on CAM (Fig. 2G). The HT-1376 CAM tumors often grew more robustly with large proliferating tumor cells in comparison to T24 CAM tumors (Fig. 2G).

Chakravarthi *et al.* used DU145 prostate cancer CAM xenografts to evaluate the role of PAICS and *de novo* purine biosynthesis in prostate oncogenesis^4^. The increasing popularity of the CAM system is supported by the fact that 7 impactful studies in the last 2 years have incorporated the CAM model to augment the mouse model of prostate cancer to investigate a range of signaling pathways and microRNA that influence invasion and metastasis^34–40^. From these recent studies, CAM xenografts have been established for all of the common human prostate cancer cell lines, including VCaP^34^^,^^35^, CWR22Rv1^36^, PC3^37^^,^^38^, LNCaP^39^, and PC-3 M-LN4^40^. In this study, we also showed that CAM xenografts could be established with human prostate cancer cell line CWR22Rv1 and C4-2, as well as the murine Myc-CaP cell line without difficulty (Fig. 2H). The large cell and nuclear morphology of prostate cancer CAM xenografts, assessed by H&E stain, were consistent with proliferative cancer cells (Fig. 2I). We and many other investigators have popularized the use of sensitive *in vivo* bioluminescence imaging (BLI) to detect small volume or disseminated prostate cancer lesions^41^^,^^42^. As shown in Fig. 2J, BLI can also detect growing CAM tumors, such as CWR22Rv1 cells that have been transduced with a firefly luciferase-expressing lentivirus.

### CAM supports the efficient engraftment of ccRCC and bladder cancer patient-derived primary cancer cells and tumor tissues

Current molecular cancer research is heavily reliant on pre-existing cancer cell lines^7^. However, many of the commonly used cancer cell lines have been cultivated in petri dishes under *in vitro* growth conditions for decades. This long-term maintenance under artificial settings raises concern over whether these cancer cell lines can still represent human cancer, and more importantly, whether findings from these cancer lines are relevant to the clinical disease^43^. Consequently, there is a strong demand to generate new primary cancer cell lines and xenografts from freshly harvested patient tumor tissues for discovery and investigative experiments ^44^. With this concern in mind, recent studies have utilized PDXs to evaluate responses to new therapies for kidney cancer^45^. For instance, Sivanand *et al.* reported intrarenal implantation of 94 tumor surgical specimens that resulted in 16 stable patient-derived grafts to assess drug response^46^. The engraftment rate of RCC PDXs in mice in recent reports is below 30%.

**Figure 3:**
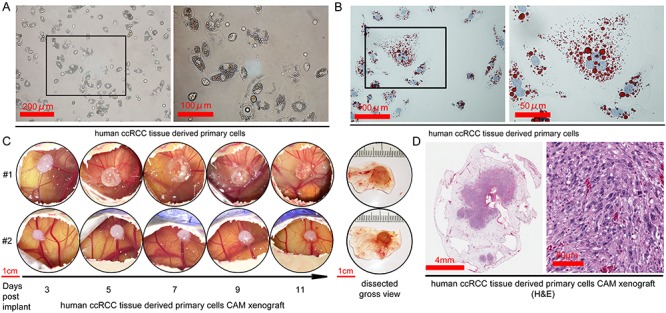
CAM can support engraftment of primary cancer cells derived from patient’s ccRCC tumor. A. The morphology of primary cancer cells derived from freshly harvested patient ccRCC tumor under phase contrast microscope. B. Oil Red O stain of tumor cells. C. The development of the primary ccRCC-derived CAM tumor from day 3 to 11 after the implantation of early passage (within 10 passages) primary cells on CAM at 2 × 10^6^ cells/egg. D. H&E stain of a CAM xenograft developed from primary ccRCC tumor cells.

In the last 2 years, we have attempted to establish renewable sources of patient-derived tumor materials from a total of 10 cases of freshly harvested ccRCC surgical samples from a single urological surgeon, Dr. Arnold Chin. Our workflow included attempts to (i) cultivate primary tumor cell lines from dissociated tumor pieces, (ii) to directly implant small tumor pieces on CAM, and (iii) to implant established primary tumor cell lines on CAM. Table 1 summarizes the successful results. To generate primary cell lines, we disassociated tumor pieces to single cells. As a representative example (Fig. 3A), the first passage of cancer cells from one case displayed epithelial morphology and contained abundant lipid droplets in the cytoplasm, verified by the lipid Oil Red O stain (Fig. 3B). We were able to establish 5 primary cell lines from the 10 cases of ccRCC. These primary cell lines remained stable for at least 5 passages *in vitro*. The CAM tumor engraftment rate using the newly generated primary cells was very high, with a successful engraftment of 4 out of the 5 primary lines. A representative case of the primary ccRCC-derived CAM tumor was shown in duplicate in Fig. 3C. Direct visualization of CAM tumor growth from day 3 to 11 can inform on the tumor vascularization process. For instance, the Matrigel (white oval chunk) of CAM tumor #2 gained a pink hue from day 7 onward, coinciding with an increase of small capillaries emanating from the tumor over time. Histological examination of the CAM tumor revealed the co-existence of cells of different sizes, as well as the majority of cells containing large nuclei, consistent with the characteristics of proliferating cancer cells (Fig. 3D).

**Figure 4:**
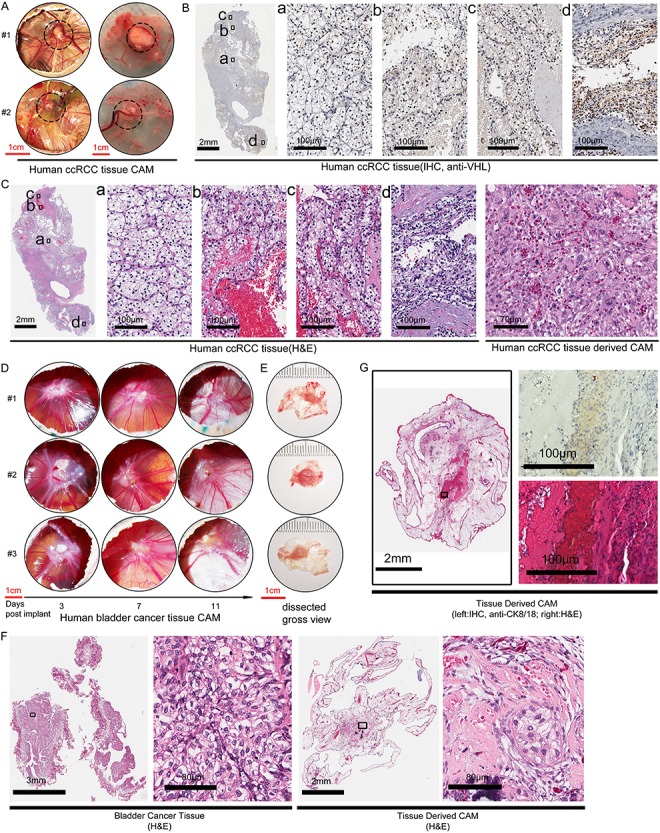
CAM ccRCC and bladder cancer xenografts derived from small pieces of patient’s tumor. A. The gross view of a representative CAM xenograft established from small pieces of a surgical sample of human ccRCC tumor. Duplicate CAM tumors of the same case at embryonic day 20 (post fertilization) was shown. Left view: *in situ* CAM with tumor above and embryo below. Right view: isolated CAM with implanted tumor. Dash circled areas indicate tumors. B. The patient’s original ccRCC tumor section assessed by anti-VHL IHC. C. H&E stain of patient’s ccRCC tumor and the corresponding CAM tumor (right panels). D. CAM xenografts from a case human bladder cancer, viewed on 3, 7 and 11 days after implantation. Triplicate engraftment of the same case was shown. E. The gross view of dissected CAM bladder cancer xenograft from D. showed the tumor size has expanded from 2-3 mm at implantation to ~ 6 mm in diameter on day 11. F. H&E stain of the patient’s bladder cancer tissue and the CAM PDX derived from it. Small foci within the CAM PDX retain cancer cell morphology similar to the patient’s tumor. G. In a different case of bladder cancer from that shown in D-F, the CAM PDX established (left panel) were subject to anti-CK8/18 human cytokeratin IHC staining (right upper panel) to identify human epithelial cells within the PDX, with its corresponding H&E stain (right lower panel).

Next, we assessed the feasibility of engrafting CAM xenografts from small pieces of fresh tumors (approximately 2 mm in diameter). Out of the 10 cases of ccRCC tumors we have attempted, the success rate of engrafting small fresh ccRCC tumor pieces was 70% (see Table 1). Figure 4A shows a representative case of CAM xenograft established from a fresh ccRCC tumor. Duplicate CAM xenografts from the same case were shown, with the left panels showing the xenografts *in situ* and the right showing the isolated xenograft with its associated CAM (Fig. 4A). A large nourishing artery can be seen coursing right of the tumor in #1, while the nourishing artery was coursing from below the CAM in tumor #2. Histological analyses of the patient’s tumor tissue by anti-VHL (Fig. 4B) and H&E stain (Fig. 4C) revealed extensive intratumoral heterogeneity amongst the 4 areas (a, b, c, d) sampled in regards to cellular morphology as well as VHL expression. Those areas contained cells with an abundance of lipid in the cytoplasm and low level of VHL expression that representing the clear cell morphology (Fig. 4B and 4C). The CAM xenograft of this case contained tumor cells that resembled those located in areas c and d of the patient’s tumor (Fig. 4C, right panel).

Bladder cancer is the second tumor type we attempted to establish primary cancer cell lines and PDXs on CAM. We have only collected fresh surgical samples of bladder cancer for 4 months as compared to over 2 years with RCC. Out of the 4 cases of surgical samples of bladder cancer harvested in the interim, we were unable to establish any primary cell lines using DMEM or RPMI-1640 media supplemented with fetal bovine serum. Previous reports have documented the need to supplement with additional growth factors, such as fibroblast growth factor (FGF)^47^^,^^48^. At this juncture, we have not optimized the culturing conditions to establish a primary bladder cancer cell line. However, establishing bladder cancer PDXs on CAM from small pieces of a tumor had been straightforward, without needing any supplement. We were able to reproducibly generate CAM PDXs from all 4 out of 4 bladder cancer cases we collected. The engraftment of PDX on CAM from a representative case was shown in triplicate in Fig. 4D. As an indication of tumor vascularization, the number of fine blood vessels coursing to and from the CAM tumor increased from day 3 to day 11 after implantation (Fig. 4D). Moreover, the size of the CAM tumor grew from 2-3 mm in diameter at the time of implantation to about 5-7 mm in diameter on day 11 (Fig. 4E). Histological examination revealed that the CAM tumor contained extensive fibrous tissues in conjunction with small foci of tumor cells that resemble the cellular morphology of the patient’s bladder tumor (Fig. 4F). In the second case of slower growing bladder cancer PDXs, we used a human cytokeratin 8/18 IHC to identify the tumor cells (Fig. 4G).

PDXs engrafted directly from patients’ tumors are extremely valuable sources of living tumor tissue for further investigation of cancer biology and pilot therapeutic studies. Although our current experience of establishing new CAM PDXs of ccRCC and bladder cancer is still quite limited in number, 10 and 4 cases, respectively, the success rate of CAM engraftment of 70%-100% is much higher than recently reported engraftment rate of PDX in mice^45^^,^^46^. The high success rate could be attributed to the richness and naïve nature of the CAM vasculature that readily vascularizes the implanted tumor cells and tissues. Furthermore, the visible nature of CAM and its short growth period are very helpful in saving time and labor in the generation of PDXs. Here, we showed CAM PDXs retained some of the cellular morphology and histological features of patients’ tumors. Confirmation that CAM PDX fully retains the characteristics of patients’ tumors will require detailed genetic and expression profiling. We are actively pursuing this line of investigation. We have found that primary ccRCC cell line and CAM tumor generated as described here retained the same genetic mutations as the patient’s tumor they were derived from (data not shown). The use of CAM PDXs as a platform to pursue a pilot therapeutic evaluation is extremely attractive, especially to fulfilling the tenet of personalized medicine. Although Vu *et al.*^49^ demonstrated the feasibility of nanoparticle-mediated drug delivery in CAM tumors, the short 10-12 day window of tumor growth and treatment on CAM would post a significant challenge to assess the traditional therapeutic endpoints such as tumor volume. We are actively investigating this critical topic.

### CAM xenograft recapitulates the metastatic behavior of mouse ccRCC model

**Figure 5:**
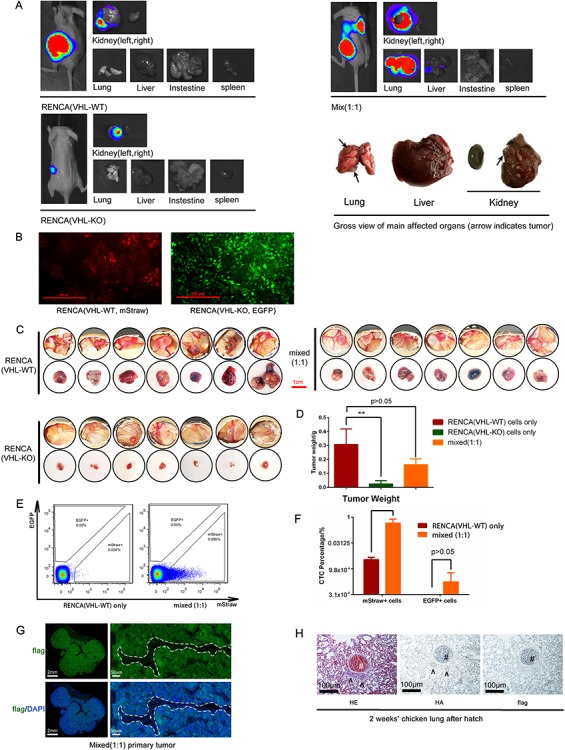
CAM xenografts recapitulate the metastatic behavior of an engineered murine ccRCC model. A. Mice were implanted orthotopically in the left kidney with either VHL wildtype (VHL-WT) RENCA cells, VHL knockout (VHL-KO) RENCA cells or a 1:1 mixture of both cells (with a total cell count of 2 × 10^6^). At 4 weeks after tumor implantation, bioluminescence imaging (BLI) was performed on each group of mice and the major organs harvested from each mouse. The enlarged gross view of the organs in the mixed tumor bearing mouse was shown in right lower panel. B. Immunofluorescence staining of VHL-WT RENCA cells (labeled with mStrawberry) and VHL-KO cells (labeled with EGFP). C. The gross *in situ* views of CAM tumor and isolated CAM tumor from each group were shown (*n* = 7 per group). D. The average tumor weight of the 3 groups of CAM tumors was shown. E. Flow cytometric analysis of circulatory tumor cell showed that mixed CAM tumor produced more (mStrawberry+) cancer cells in the blood of chick embryo. F. RT-PCR analysis confirmed that VHL-WT (mStrawberry+) cells were the predominant circulating tumor cells. G. Immunofluorescence stain of the FLAG-tagged tumor cells (green) that invaded into vasculature. The CAM tumor cells (green) could be distinguished from avian stromal cells and nucleated red blood cells. The areas within the white dash line indicate the blood vessels and the white arrow indicates a nucleated chicken red blood cell. H. CAM tumors were established with a 1:1 mixed of VHL-WT cells (HA tagged) and VHL-KO cells (FLAG tagged) and embryos were allowed to hatch and grow for additional 2 weeks. Immunohistochemical analyses of lung sections from the 2-week old chick were shown. Arrows indicate two metastatic lesions in lung and # indicates a big blood vessel in the chicken. (**: *p* < 0.01)

Metastasis to lungs is a frequent and deadly manifestation of ccRCC in the clinic. Unfortunately, the lack of clinically relevant spontaneous metastatic ccRCC models has slowed the understanding and the development of effective treatment for this disease. We created a novel metastatic ccRCC model by CRISPR-mediated VHL gene deletion in the murine RENCA line^26^^,^^27^, and established the parental VHL wildtype (VHL-WT) and VHL knockout (VHL-KO) RENCA cells. We have observed that VHL deletion leads to epithelial-mesenchymal transition (EMT) of VHL-KO cells and dramatic slowing in proliferation *in vitro*^26^^,^^27^. As shown in Fig. 5A, VHL-WT cells grew well after implanted into the kidney but did not produce metastasis in distant organs. Replicating their *in vitro* phenotype, the EMT+ VHL-KO cells grew poorly in the kidney (Fig. 5A). Strikingly, a 1:1 mix of VHL-WT and VHL-KO cell-implanted tumors not only grew well in the kidney, but also produced rampant metastasis in the lung and, to a lesser degree, in the liver (Fig. 5A). These results suggested that an intriguing cooperative mechanism of metastasis is at play, in which the poorly-proliferative EMT+ VHL-KO cells induce the metastatic potential of non-EMT VHL-WT cells^26^^,^^27^.

**Table 1:** A summary of CAM xenograft engraftment from different cell or tissue sources of kidney, bladder or prostate cancer.

	Kidney cancer (ccRCC)	Bladder Cancer	Prostate Cancer
Cell line	RENCA, ACHN	HT-1376, T24	CWR22v1, C4-2, Myc-CaP
Primary tumor cells	YES, 4 out of 5 cases	Not yet tried^*^	Not yet tried
Tissue chunks	YES, 7 out of 10 cases	YES, 4 out of 4 cases	Not yet tried
Xenograft integrity	Good	Moderate, small foci of tumor with extensive fibroblasts	Good for cell lines
Advantages as compared to mouse model	Shortened period of vascularization (~2 days) Shortened period of overall tumor growth with comparable size In general, CAM tumor with ~2x10^6 tumor cells can grow to 1 cm in diameter in 10-11 days Great saving in cost (~$1 for each fertilized egg) in comparison to mouse (>$100 for each immunocompromised mouse) Tumor growth visible to naked eye
Disadvantages as compared to mouse model	Difficult to achieve significant tumor expansion with slow growing tumor cells or tumor tissues in the short 10-11 days growth period allowed in CAM Challenging to assess treatment response in 11 days Difficult to detect metastasis in chick embryo organs due to the short time period of growth and different circulation pattern		

^*^Unable to recover primary tumor cells from surgical tissues with RPMI-1640 or DMEM media supplemented with 10% FBS.

Here, we strive to assess whether the CAM xenograft can also recapitulate the growth and metastatic behavior of our VHL-KO and VHL-WT RENCA model^26^^,^^27^. To facilitate the tracking of these two clonal purified cell lines *in vivo,* we marked them by lentiviral transduction, VHL-WT cells with the mStrawberry fluorescence protein and VHL-KO cells with the EGFP (Fig. 5B). These two cancer cell lines were also tagged with FLAG antigen to aid their identification by histological analyses. The CAM of 21 fertilized eggs were implanted with either VHL-WT cells only or VHL-KO cells only or a 1:1 mixture of these two (*n* = 7 per group). The growth rate of the CAM tumors was similar to their mouse counterparts^26^ (Fig. 5A). The VHL-WT and mixed cell group of CAM tumors grew well, while the CAM tumors of VHL-KO group grew poorly. These findings were confirmed by measurement of tumor size (Fig. 5C) and weight (Fig. 5D). To assess tumor cell escape into circulation, the first intravasation step of metastasis, we analyzed circulatory tumor cells by flow cytometry. As shown in Fig. 5E and 5F, the presence of VHL-KO cells in the mixed tumor greatly enhanced the number of VHL-WT cells in circulation. Also, it confirms that the number of cancer cells that escaped into circulation in the mixed CAM tumor was much higher than that in the CAM tumors with VHL-WT cells only (Fig. 5E), and the majority of circulatory tumor cells was mStrawberry+ VHL-WT RENCA cells (Fig. 5F). To examine the vascular invasion of tumor cells in the CAM xenograft, we used immunofluorescent staining with an anti-FLAG antibody to identify tumor cells at the tumor and vessel junction. Figure 5G shows the presence of tumor cells (FLAG+) interspersed with chicken nucleated red blood cells (white arrow) within a blood vessel (demarcated by the dashed line).

Due to the short growth period on CAM before hatching and the decreased blood perfusion to the uninflated chicken embryo lung, detecting metastasis in the lungs of chicken embryo was expected to be very challenging. To overcome these limitations, we obtained approval from the Institutional Animal Care and Use Committee (IACUC) to extend the analyses of distant metastases in hatched chickens. In a separate experiment, we implanted a CAM tumor with 1:1 mixed VHL-WT and VHL-KO RENCA cells that were marked by HA-tag and flag-tag, respectively. The hatched chicks that bore CAM tumors were grown for 2 additional weeks before euthanasia and tissue analyses (Fig. 1). This time extension enabled the cancer cells to establish small metastatic nodules in the chicken lung, as visualized by H&E stain (Fig. 5H). A majority of the tumor cells in the metastatic lesion was the HA-tagged VHL-WT cells. The flag-tagged VHL-KO cells were difficult to locate (Fig. 5H). This finding is highly consistent with what we observed in the mouse model (data not shown). Importantly, the avian CAM tumor model can reproduce the preferential homing of ccRCC tumor cells to the lungs, which is observed in clinical disease and our mouse model^50^.

In this study, we demonstrated that CAM is an efficient system to establish xenografts from either pre-existing cancer cell lines, primary cancer cell lines or small tumor pieces from patient-derived ccRCC or bladder tumors. Table 1 summarizes successful CAM xenografts we have attempted in the last 2 years, as well as the advantages and disadvantages of CAM in comparison to the mouse model. The CAM tumor model is now well-accepted by the scientific research community, supported by the fact that the number of publications involving CAMs has increased 10 folds from 2000, and the findings are often published in the most prestigious journals ^5^^,^^24^^,^^51^. Given the high efficiency of PDXs engraftment on CAM in a short 10-day period, it holds great promise as an *in vivo* platform to pursue pilot drug screening on individual patient’s tumor. Although many challenges remain unsolved to achieve the ultimate goal of precision individualized medicine with CAM, it is proven to be a convenient *in vivo* system to accelerate the discovery of critical molecular mechanism in cancer biology, such as the lethal metastatic disease.
